# Evaluating the growth and water use efficiency of chili pepper ‘Tanjung’, ‘Unpad’, and ‘Osaka’ using SFM1 sap flow in agro-environment

**DOI:** 10.1038/s41598-026-39053-x

**Published:** 2026-02-27

**Authors:** Kusumiyati Kusumiyati, Farhan Ahmad, Mochamad Arief Soleh, Meilinda Rahayu Putri, Nurul Khania Ariani Kusdinar, Ian Sebastian, Ristina Siti Sundari

**Affiliations:** 1 Department of Agronomy, Agricultural Faculty, Universitas Padjadjaran, Jl, Bandung-Sumedang km 21 Jatinangor, Sumedang, West Java Indonesia; 2 PT. Labodia Prima, Jakarta, Indonesia; 3 Department of Agribusiness, Faculty of Agriculture, Universitas Perjuangan, Jl. PETA No. 177, Tasikmalaya, West Java Indonesia

**Keywords:** Environmental stress, Plant physiology, Plant-water relation, Resource optimization, Water productivity, Ecology, Ecology, Environmental sciences, Plant sciences

## Abstract

Water use efficiency in horticultural crops is necessary for sustainable agriculture, especially under changing micro climate conditions. This study assesses the water use efficiency (WUE), growth, and physiological performance of three chili cultivars (Tanjung, Unpad, and Osaka-3) under various conditions of agro-environment. The experiment was carried out from December 2023 to May 2024 at Universitas Padjadjaran in Indonesia, using a factorial randomized complete block design with 432 plants spread among four conditions of agro-environment: greenhouse, rain shelter, screen house, and open field. Plants were given nutrient solutions in varied quantities based on evapotranspiration (ETc) in four conditions of agro-environment. a Sap Flow Meter (SFM1) with the Heat Pulse Velocity technique measure volume of nutrient solution for watering the plants which was predicted using a soil water balance model. The results revealed interaction and significant effect of cultivar and four conditions of agro-environment that influenced water intake, sap flow, and growth. Osaka had the highest water sap velocity, indicating intense transpiration, particularly in the screen house and open field. Tanjung performed best in the screen house, with the maximum WUE (2.0 g/L) and absolute growth rate (> 0.60 cm/day), despite the low water usage. Correlations between sap velocity and water consumption for absolute growth rate (AGR) was 0.68* and WUE was 0.59. These findings emphasize the significance of controlled conditions of agro-environment designs to maximize water use efficiency and maintain chili productivity.

## Introduction

Condition of agro-environment has the potential to threaten conventional crop production systems^1^. Rising temperatures may impact crop development and performance unless further precautions are taken, including the emergence of abiotic disorders^2^. Weather and climatic conditions might impact production efficiency and chili farming income^3^. Chilies are sensitive to environmental conditions, soil type, and fertilization. Growing chili pepper in greenhouses with controlled conditions in places with diverse climates is preferable^4^. Biotic and abiotic stress affect the productivity and quality of vegetables cultivated in the open field^5^. Protected cultivation can mitigate both biotic and abiotic stress^6^.

Plants frequently experience water deficiencies in the soil and atmosphere throughout their life cycle^7^. Stomatal activity indicates healthy growth in chili plants^8^. Stomata are essential organs of vascular plants that regulate gas and water exchange between the inside of the plant and the external air^9^. Water scarcity is a common environmental factor that impedes plant growth^10^. The efficient water use resources and scarcity are becoming more serious issues in global agriculture, particularly in areas with unpredictable climate and area of water scarcity^11^. Water availability primarily influences leaf and root growth, and also photosynthesis^12^. Because of their high water content (70–90%), postharvest chilli pepper fruits tend to experience extensive water loss and other metabolic activities^13^. Long-term light intensity has a substantial influence on chili flowering^14^. The vegetative growth necessitates sufficient nourishment in water, which is required for the initial development of red chili flowers and fruits^15^. Chili are highly vulnerable to drought stress due to their extensive leaf surface transpiring and higher stomatal conductivity^16^.

Plant cultivation is an essential phase for achieving optimal plant growth^17^. The greenhouse is a system for cultivates plants and lessen insect and disease attacks on plants while allowing for easier control of micro climate and soil conditions^18^. Screen and shade nets have pivotal functions in a controlled environment and micro climate condition^19^. Screens can be utilized in greenhouses to optimize temperature control at the expense of energy for heating and cooling^20^. Screens are fitted automatically in greenhouses when environmental thresholds are crossed^21^. Open field farming, commonly used, has various drawbacks for maximizing chili pepper yield compared to a controlled agro-environment^22^,^23^. Unpredictable rainfall, temperature fluctuations, and elevated pest levels commonly result in variable growth and reduced yield consistency^24^. Conditions in open fields also obstruct accurate water management, complicating irrigation optimization and evaluating physiological characteristics^25^,^26^.

The research intends to assess the water use efficiency and growth performance of chili plants under various agro-environmental conditions utilizing the SFM1 Sap Flow system at field capacity, hence providing insights into appropriate irrigation techniques for sustainable chili pepper cultivation.

## Materials and methods

### Research site & materials

The experiment was conducted from December 2023 to May 2024. The experiment used three chili pepper cultivars (‘Tanjung’, ‘Unpad’, and ‘Osaka-3’) grown at the Bale Tatanen Research field, in the Faculty of Agriculture, Padjadjaran University, Jatinangor, Sumedang Regency, at an elevation of 685 m above sea level in different agro-environments (greenhouse, rain shelter, screen house, and open field).

The materials that used were plastic containers (40 × 40 cm), planting media weighing 1.2 kg made of biochar and cocopeat at 2:1, water, organic and chemical fertilizers, biopesticides, bioherbicides, bio fungicides, UV plastic sheeting (200-micron), a 50-mesh screen net. The equipment used for the experiment were a Sap Flow Meter (SFM1), thermo recorder, lux meter, anemometer.

### Agro-environments

**Fig. 1:**
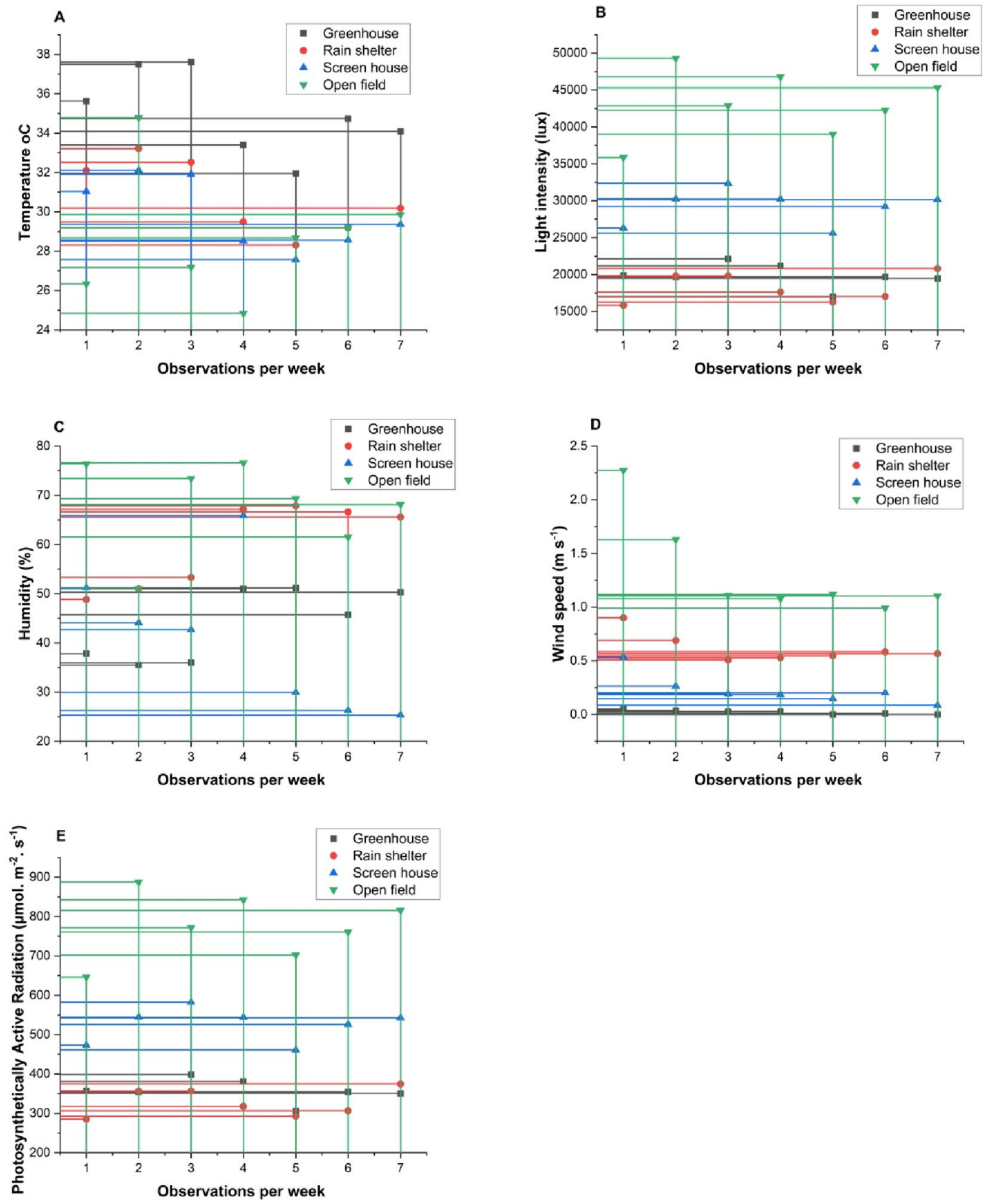
(**A**-**E**). Microclimates differentiation in different agro-environments.

Planting occurred in four distinctive agro-environments: greenhouse, rain shelter, screen house, and open field, each with microclimatic parameters such as temperature, humidity, light intensity, photosynthetically active radiations, and wind speed (Fig. 1).

The greenhouse construction measured 24 m long, 17 m wide, and 6 m tall. Its roof was made of 200-micron UV-stabilized plastic, and the walls were lined with a 50-mesh screen net to control airflow and pest entrance. The rain shelter had a 200-micron UV plastic roof and was 18.5 m in length, 5 m in breadth, and 3.5 m high. The screen house measured 15 m long, 3.5 m wide, and 2.8 m high, with just the top covered by a 50-mesh screen net.

### Watering method

Watering was done daily using a mixed-AB nutrient solution with the following composition: Solution A contains NPK, calcium, magnesium, sulphur, and iron. Solution B contains manganese, boron, copper, zinc, and molybdenum micronutrients. Solution A and B, a total of 2 L, are dissolved in 96 L of water. The nutrient solution was given above the planting medium. The difference in watering amount was established two weeks after the transplanting of the plants.

The soil water balance equation estimates the irrigation volume based on plant evapotranspiration:

1 $${\mathbf{ETc}}\, = \,{\mathbf{P}}\, + \,{\mathbf{I}} - {\mathbf{R}} - {\mathbf{D}} - ({\mathbf{W1}} - {\mathbf{W2}})$$

Note: 

ETc: Evapotranspiration (mm).

P: Precipitation (mm).

I: Irrigation (volume of water given) (mm).

R: Run off (Surface flow) (mm).

D: Drainage (Percolation) (mm).

W_1_: Media weight (g) after applying water till field capacity.

W_2_: Weight (g) of media on following day.

Three plant samples were collected from each location to determine total evapotranspiration (ETc). Each plant sample was fitted with a water storage container under the polybag to determine the quantity of percolation (D) caused by watering. The weight of the planting medium (W) was determined by weighing both the planting medium and the plants. Daily evapotranspiration measurement was carried out every day, such that three samples of plant medium were taken in every treatment of the cultivar, and water was supplemented to field capacity, with the weight of media and percolated water noted. Plant medium was weighed the next day to get the weight change (evapotranspiration). The measured difference was assumed to be evapotranspiration from the given plant medium and was assigned the value 100% ETc.

### Sap flow meter (SFM1) processing

**Fig. 2:**
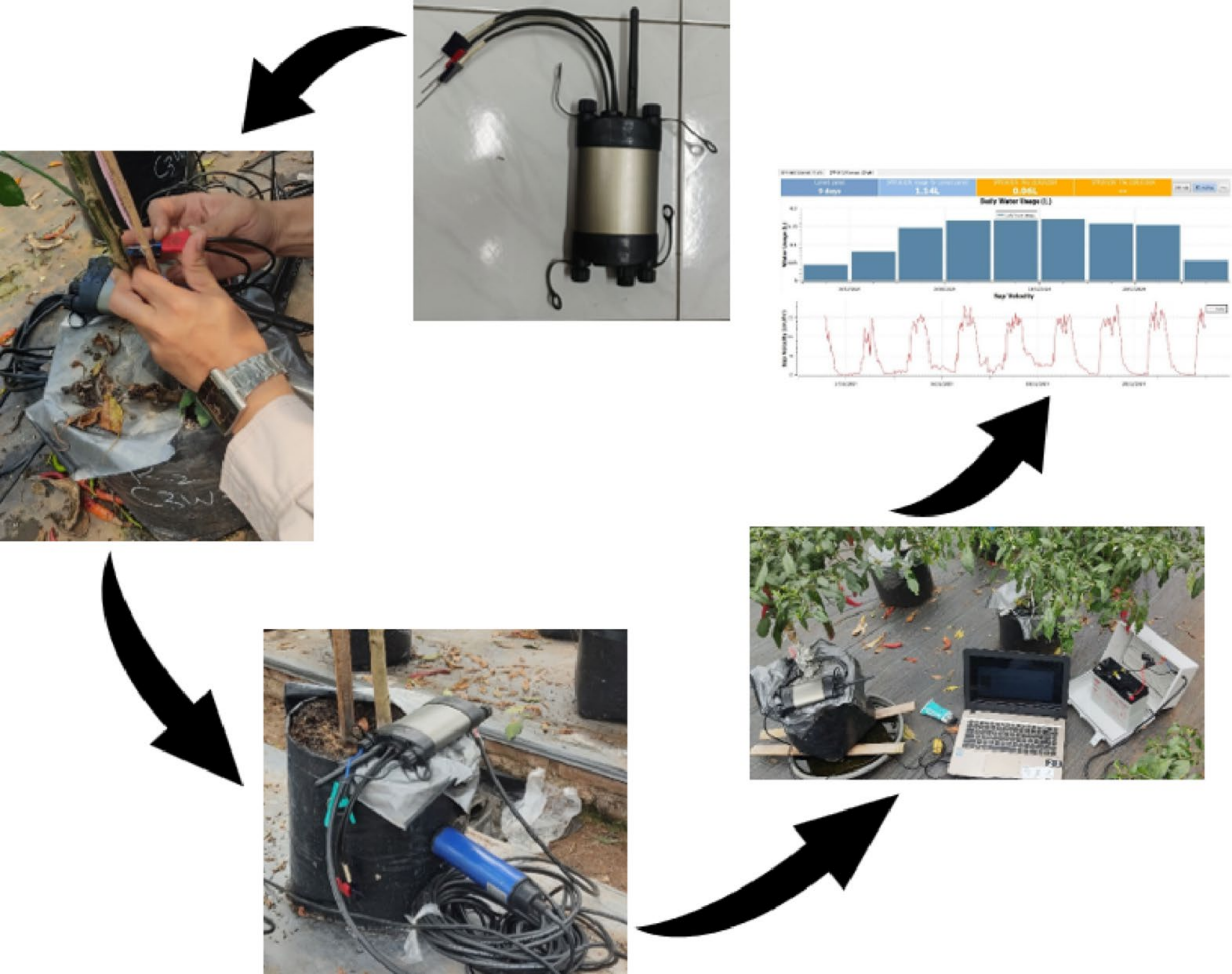
Sap flow meter (SFM1) operation and output data collection process.

The Sap Flow Meter (SFM1) uses the Heat Pulse Velocity (HPV) method to measure plant sap flow (Fig. 2). The device consists of a central heating probe and two temperature-sensing probes along the stem above and below the heater. During measurement, the central probe emits a brief heat pulse, and the sensors detect temperature changes as heat is transferred through the flowing sap. The time required for the heat to transfer between the probes is used to calculate the sap flow rate, which is directly proportional to the water flow within the plant. The SFM1 device is connected to a data logger that continuously records the temperature difference and estimates the sap flow in real time. Before installation, the bark must be carefully removed and the probe inserted into the trunk at a specific depth to ensure reliable readings. Batteries or solar panels power the meter and require regular calibration based on the trunk diameter and wood characteristics. The logger collects daily sap flow rate and water consumption data, which are then evaluated to determine how much water the plant consumes under various environmental and treatment conditions.

### Experimental design

The experiment design using Factorial Randomized Complete Block Design was developed using the linear model equation as follows:

2 $${\mathbf{Y}}_{{{\mathbf{ijk}}}} = {\text{ }}{\mathbf{u}}{\text{ }} + {\text{ }}{\mathbf{C}}_{{\mathbf{j}}} + {\text{ }}{\mathbf{A}}_{{\mathbf{k}}} + {\text{ }}({\mathbf{AC}})_{{{\mathbf{ij}}}} + {\text{ }}{\mathbf{r}}_{{\mathbf{l}}} \, + \,{\mathbf{\varepsilon }}_{{{\mathbf{ijk}}}}$$

**Y**_**ijk**_ = is the observed value of the response variable;

**u** is the overall mean;

C_j_ is the fixed effect of the J^th^ cultivar;

**A**_**k**_ is the fixed effect of the K^th^ agro-environment;

**(CA)**_**jk**_ represents the interaction effect between cultivar and agro-environment;

**r**_**i**_ is the effect of the I^th^ replication (block);

**ε**_**ijk**_ is the random experimental error.

Note: C=Cultivars, A=Agro-environments, AC=Interaction of agro-environments x cultivars.

i = 1, 2, … a.

j = 1, 2, … b.

k = 1, 2, … c.

l = 1, 2,… k.

The research hypothesis:

H_0_: C_1_ = C_2_ = … = C_n_ = 0; H_1_: at least one of C_i_ ≠ 0.

H_0_: A_1_ = A_2_ = … = P_n_ = 0; H_1_: at least one of P_i_ ≠ 0.

H_0_: (CA)_ij_ = 0 for all i and j; H_1_: at least one of (CG)_ij_ ≠ 0.

If the interaction effect is significant, hypothesis testing of main effects is unnecessary since the interaction takes precedence over the main effects. If the interaction effect is insignificant, hypothesis testing of main effects is necessary. Post-hoc test determines the best treatment for factor levels. Tukey’s Honestly Significant Difference (HSD) test is often employed. Tukey’s HSD test steps are as follows.

- Order treatments mean accordingly.

3 $$\:\boldsymbol{\omega\:}={\boldsymbol{q}}_{\propto\:}(\boldsymbol{p},\boldsymbol{v})\sqrt{\frac{\boldsymbol{S}}{\boldsymbol{r}}}$$

Where: p = treatment amount = t, v = error degree of freedom, r = replication amount, α = confident level, q_α_(p, v) = critical value that obtained from t-student table.

### Data analysis

The data was analyzed using SPSS software, with variance analysis (ANOVA) used to examine the significance of treatments, followed by Tukey’s post hoc test to estimate mean differences at a 5% significance level. Additional statistical analyses were carried out with Statistics 8.1 to facilitate comparison evaluations. Origin Lab and Cloud TUTU software was used to create graphical visualizations, data plots and managed experimental datasets while facilitating collective data analysis.

## Results and discussion

The comparative impacts of various agro-environments and chili pepper varieties on important growth and water-use parameters (Table 1). In particular, it outlines differences in absolute growth rate, water use efficiency, daily water consumption, and sap flow, emphasizing how cultivation conditions and genetic diversity affect plant physiological performance.

**Table 1:** Effect of agro-environments and cultivars on growth and water use efficiency of Chili pepper.

Treatments	Observations			
	Absolute growth rate (cm *p*^− 1^ day^− 1^)	Water use efficiency (g/L)	Daily water usage (mL)	Sap velocity (cm/hr)
Agro-environments				
Greenhouse	0.44^a^	1.07^b^	336^a^	22^a^
Rain shelter	0.44^a^	1.37^a^	230^a^	21^a^
Screen house	0.53^a^	1.52^a^	550^a^	24.5^a^
Open field	0.41^a^	0.94^b^	446^a^	25.3^a^
Cultivars				
Tanjung	0.49^a^	1.46^a^	445^a^	22.5^a^
Unpad	0.39^a^	1.05^b^	322^a^	21.9^a^
Osaka	0.47^a^	1.16^b^	405^a^	25.2^a^

Means with different lettering (^a, b, c^) showing significant (*P* < 0.05) effect among treatments.

## Results

**Fig. 3:**
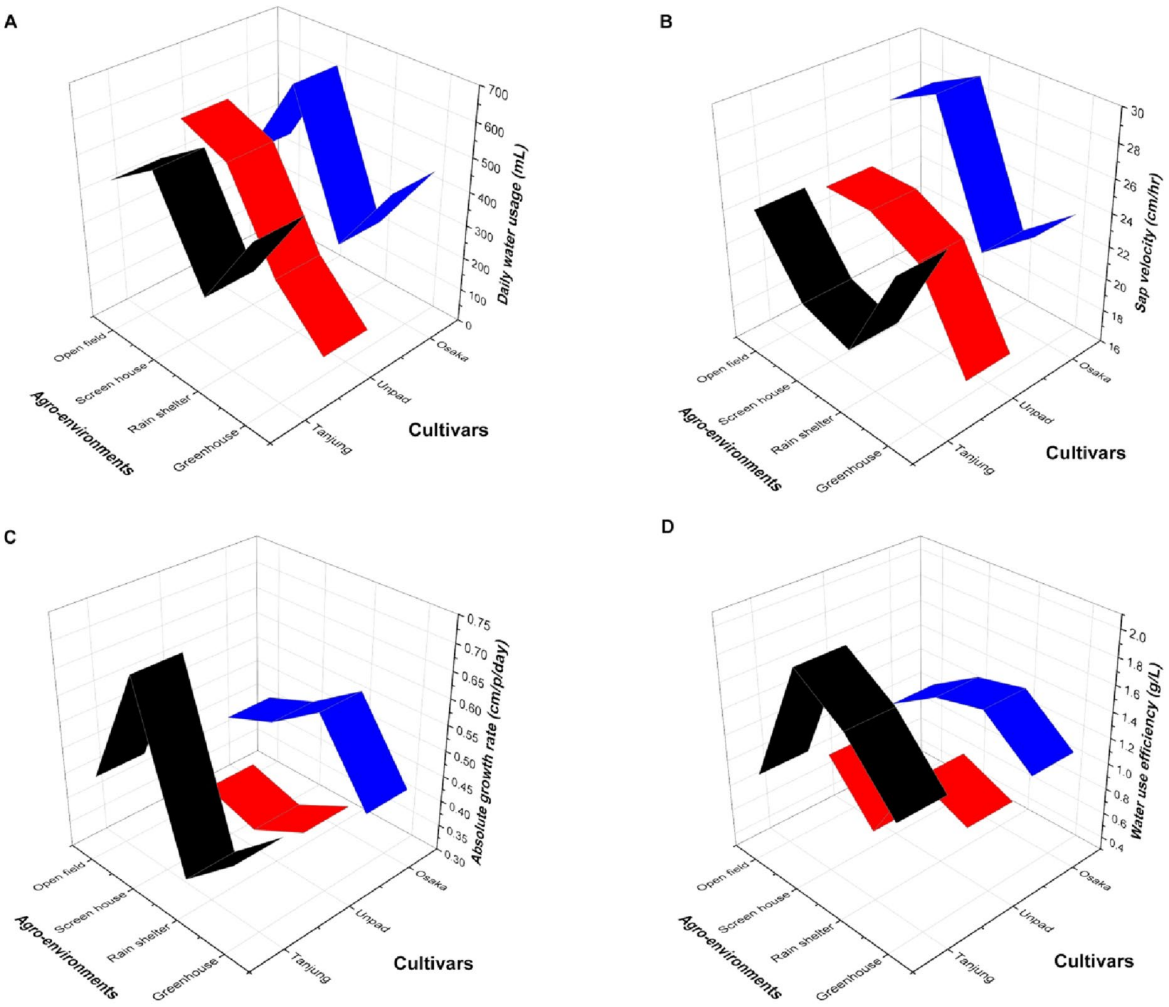
(**A**, **B**, **C**, **D**). Effect of cultivars and agro-environments on daily water usage, sap velocity, absolute growth rate, and water use efficiency.

The figure (Fig. 3A) shows the three chili cultivars, Osaka, Unpad, and Tanjung, for daily water intake (mL) in four agro-environments: greenhouse, rain shelter, screen house, and open field. The results show significant correlations between environmental conditions and cultivar performance regarding water intake. Among the cultivars, Osaka consumed the highest amount of water per day, particularly in the screen house and open field environments, where it consumed approximately 600 mL per day. Unpad trailed, exhibiting a comparatively high-water need in exposed conditions and excessive water use in the open field but still less than Osaka. Tanjung had the lowest daily water consumption, especially in the screen house and greenhouse conditions.

Cultivar Osaka always exhibited greater sap velocity, the highest values in the screen house and the open field suggesting very active xylem transport of both semi-controlled and open field conditions (Fig. 3B). It is in keeping with Osaka’s reported vigorous growth, possibly due to high transpiration and nutrient streaming. Unpad, while not as tall as Osaka, had reasonably good sap velocities in all conditions with clear strength in the open field and screen house. Tanjung recorded the lowest sap velocity, especially under rain shelters and screen houses. This reduced velocity could indicate reduced transpiration rates or a water-saving method for water usage, possibly with the benefit of increased resilience in more humid or enclosed conditions but at the cost of reduced growth activity. Interestingly, the greenhouse environment yielded lower sap velocities across cultivar Unpad, indicating the possible influence of greater humidity and lower vapor pressure deficit in limiting transpiration-driven sap flow.

The plot shows the interaction between cultivars and agro-environments on chili plants’ absolute growth rate (AGR) (Fig. 3C). Among all combinations, the Tanjung cultivar in the screen house had the highest AGR, greater than 0.60 cm/day, indicating a high positive growth response under protected and regulated conditions. On the other hand, the Tanjung cultivar in the rain shelter exhibited a minimum AGR below 0.35 cm/day as an indicator of low adaptability to the particular environment. The AGR values for the Osaka cultivar remained relatively consistent for all environments, with the highest number in the rain shelter and reducing slightly in the open field and the greenhouse, indicating moderate adaptation. The Unpad cultivar had lower AGR throughout all Agro-environments, i.e., reduced overall growth rate. Screen house and greenhouse conditions were advantageous for growth among cultivars, but rain shelter and open fields were not so favourable, particularly for Tanjung.

The figure illustrates the influence of agro-environments and cultivars on water use efficiency (WUE) (Fig. 3D). Tanjung cultivar in the screen house captured the highest WUE of up to 2.0 g/L, exhibiting greater capacity in water conversion into biomass under controlled conditions. For instance, the same cultivar has a marked decline in WUE in rain shelter and greenhouse environments, indicating sensitivity to less-than-ideal or humid conditions. The Unpad cultivar, represented in red, consistently shows lower WUE values in all the conditions, indicating poor water use adaptation or efficiency. The Osaka cultivar has moderate performance, with the optimum WUE in the screen house and rain shelter and relatively consistent but low values in the open fields and greenhouse.

### Clustering and correlation coefficients

**Fig. 4:**
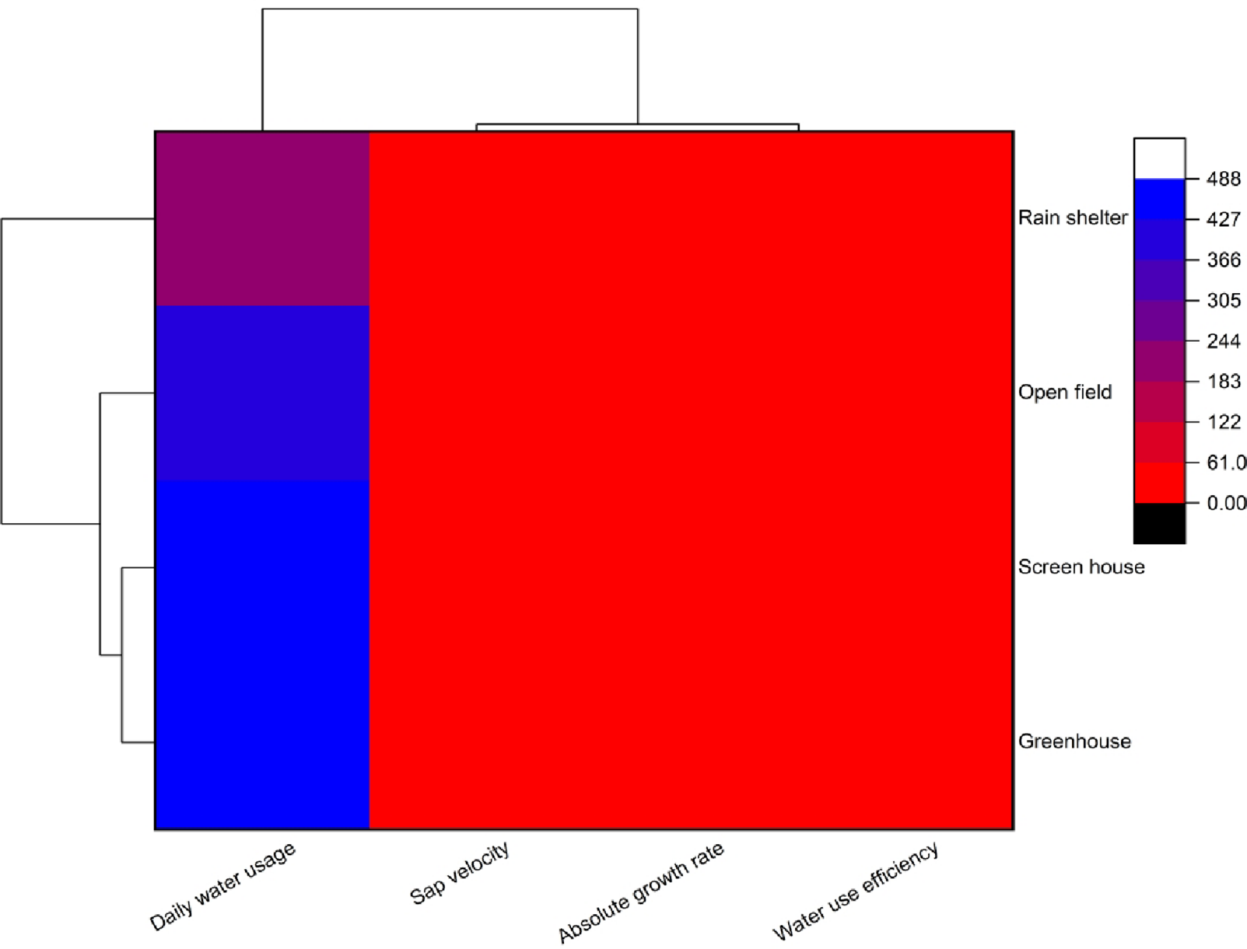
Cluster heatmap of the studied parameters.

The clustered heatmap shows the relation between four physiological characteristics—daily water use, sap velocity, absolute growth rate, and water use efficiency—and four agro-environments: greenhouse, screen house, rain shelter, and open field (Fig. 4). The color gradient is from red (lower values) to blue (higher values), allowing visual comparison between treatments. Greenhouse and screen house environment have the most significant daily water demand, possibly due to increased transpiration and perfect plant growth conditions. In contrast, the rain shelter and open field need significantly less water, with water consumption. All agro-environments have low values (red) for sap velocity, absolute growth rate, and water use efficiency throughout, indicating that these characteristics do not vary significantly across environments. Hierarchical clustering in the heatmap groups environments into clusters depending on their similarity in overall trait expression. Greenhouses and screen houses cluster very close together, indicating similar physiological profiles, specifically regarding water requirements.

**Fig. 5:**
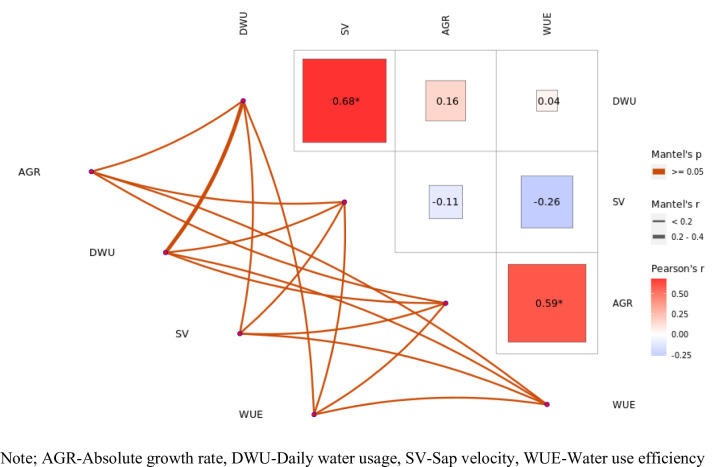
Correlation coefficient (Matel’s and Pearson’s correlation of the observations).

While open field and rain shelter form their cluster, showing a common tendency of reduced daily water consumption. The clustering nature shows daily water consumption as the most discriminating variable between agro-environments, while other factors have a secondary role in differentiation. The graph depicts a correlation matrix and network graph that illustrate the correlations among variables. The right-hand side heatmap illustrates Pearson’s correlation coefficients, where positive correlations are depicted in red and negative correlations in blue (Fig. 5). Remarkably, there is a very high positive relationship between daily water usage (DWU) and sap velocity (SV) (*r* = 0.68*) and between absolute growth rate (AGR) and water use efficiency (WUE) (*r* = 0.59*), which implies that greater water consumption is associated with greater sap velocity. On the other hand, greater growth is associated with greater water use efficiency.

There are weak and statistically non-significant correlations between DWU-AGR (*r* = 0.16) and SV-AGR (*r* = -0.11). The left network plot displays these interactions in edges of varying thickness to reflect Mantel’s r and the color intensity to represent Mantel’s p-value. Thicker and darker lines suggest stronger relations, although the majority are insignificant. The graph depicts the significant correlations and demonstrates that there are no strong links between some of the variables. This matrix helps to describes direct and indirect correlations between physiological and water attributes in the system under study.

## Discussion

Cultivation in humid subtropical climates highlights that microclimate—particularly VPD and radiation—determines transpiration and irrigation needs, with sheltered structures decreasing sap flow and water usage compared to open conditions because of diminished evaporative gradients^27^. Reducing the water supply is not suggested in chili production^34^ since it can lessen yield and quality by limiting irrigation to 80%^35^. The hour needs to achieve maximum yield per unit of water^36^. Water stress below field capacity decreased stomatal conductance and photosynthesis^37,38^. When plants get sufficient water to fulfill their needs, transpiration efficiency is enhanced^39^, lessening water loss through deep percolation and evaporation^40^. The reduced sap velocities in the greenhouse indicate humidity-related limitations on transpiration^28^. Elevated sap flow and water absorption in semi-controlled and open environments suggest a high-conductance phenotype, probably associated with robust growth and a transpiration-driven nutrient transport strategy^29^. Elevated sap flow and absorption, resulting from increased VPD, can promote growth when water is abundant, but may reduce WUE during stress^19,30^. The elevated WUE demonstrates how efficient water use, combined with sufficient light and moderate VPD, can enhance biomass conversion for each unit of water^31,32^. Reduced WUE in different environments corresponds with findings that specific cultivars need more accurate irrigation systems and plant densities to attain satisfactory efficiency^33^.

Precise irrigation use water effectively for each crop^41^. Water balance in the plant compensate for water loss due to evaporation throughout the day caused by flood without causing excess and insufficient watering^42^. A 40% watering with maximum allowable depletion resulted in maximum water use efficiency for chili production^43^. Watering plants to 20% − 50% of their maximum root depth is sufficient to meet water requirements at vulnerable stages of plant growth^44^. Overwatering directly influence crop yield and phytonutrient quality^45–47^. The plants can use adequate moisture for physiological processes at sufficient watering^35^ without experiencing the stress of water deficiency or oxygen deficiency due to waterlogging^47^,^48^. Chili plants watered to field capacity exhibited a 20–30% better WUE under controlled microclimates than open field conditions^49–51^.

Most biotic and abiotic factors affect open-field crops, reducing crop yield^24^. Cultivating crops in controlled environments reduces stress and produces better crop productivity^4^. Microclimate structures modify ambient conditions of temperature, light intensity, relative humidity, significantly influencing plant physiology^52^. Chili plants grown in a screen house possessed higher plant height, chlorophyll content, and fruit weight^53^. Moderate temperature and reduced evapotranspiration in the screen houses favour an effective photosynthetic rate and reduced water stress^25,54^. Microclimates of intermediate temperatures retard soil water loss^55^, allowing water to linger in the root zone for an extended duration^56^. Microclimates minimizes irrigation frequency without affecting plant water status and physiological processes^57^. Temperature indirectly controls crop development by balancing the rates of photosynthesis and respiration^58^. Temperature affects plant growth and physiology, directly or indirectly impacting crop yield^59,60^. Watering volume reduces water stress and allows for controlled evapotranspiration in greenhouse^61^, resulting in higher rates of photosynthesis and more even vegetative growth^62^. Open fields compensate for higher evapotranspiration and fluctuating climatic conditions by better meeting the plants’ water needs^6^. Controlled environments indicate that water deficit and heat stress can affect crop growth, development, and yield^63^,^64^. Using drought-tolerant chilies in the tropics can reduce production costs while increasing plant productivity^12^. Chili peppers should ideally be planted in a niche that suits the ever-increasing needs for maximum growth and productivity like altitude, plant medium, and microclimate^65^.

## Conclusion

The cultivar Osaka took up the most water daily—600 mL/day—and sap velocity, particularly in the screen house and open field, representing high transpiration and good xylem transport. Unpad had low water uptake and sap velocity but always had the lowest WUE in all agro-environments, indicating inefficiency in the conversion of water to biomass. Despite its low daily water usage and sap velocity, Tanjung cultivar had the maximum WUE value of 2.0 g/L under the screen house conditions and maximum absolute growth rate (AGR) of more than 0.60 cm/day in the same agro-environment. Tanjung performs best in controlled environments, balancing water conservation with biomass production. Environmental evaluation showed that the screen house offered the most suitable conditions, increasing AGR and WUE, particularly for Tanjung. Cluster and correlation analyses established that greenhouse and screen house environments support higher water requirement and physiological response, as well as positive correlations between sap velocity and water consumption daily and AGR and WUE.

## Data Availability

The data will be made available on reasonable request to corresponding author (kusumiyati@unpad.ac.id) if required.
